# *trans*-(5,7,7,12,14,14-Hexamethyl-1,4,8,11-tetra­aza­cyclo­tetra­deca-4,11-diene-κ^4^ *N*,*N*′,*N*′′,*N*′′′)bis­(nitrito-κ*N*)cobalt(III) perchlorate hemihydrate

**DOI:** 10.1107/S1600536811042784

**Published:** 2011-10-22

**Authors:** Tapashi G. Roy, Babul C. Nath, Khadija Begum, Seik Weng Ng, Edward R. T. Tiekink

**Affiliations:** a Department of Chemistry, University of Chittagong, Chittagong-4331, Bangladesh; b Department of Chemistry, University of Malaya, 50603 Kuala Lumpur, Malaysia; c Chemistry Department, Faculty of Science, King Abdulaziz University, PO Box 80203 Jeddah, Saudi Arabia

## Abstract

The asymmetric unit of the title Co^III^ complex, [Co(NO_2_)_2_(C_16_H_32_N_4_)]ClO_4_·0.5H_2_O, comprises two complex cations, two perchlorate anions and a water mol­ecule of crystallization. The Co^III^ atoms exist within distorted octa­hedral N_6_ geometries defined by four N atoms of the macrocycle ligand and *trans*-N atoms derived from the nitrite anions. Systematic variations in the Co—N bond lengths are correlated with the presence of intra­molecular N—H⋯O(nitrite) hydrogen bonds. In the crystal, water-O—H⋯O(perchlorate) hydrogen bonds, involving one of the independent perchlorate anions only, lead to supra­molecular chains along the *b*-axis direction. The three-dimensional architecture is consolidated by numerous C—H⋯O inter­actions. The crystal studied was a non-merohedral, racemic twin.

## Related literature

For background to macrocycles and for related structures, see: Roy *et al.* (2006 ▶); Hazari *et al.* (2008 ▶). For the synthesis, see: Curtis & Hay (1966 ▶); Bembi *et al.* (1984 ▶). For additional geometric analysis, see: Spek (2009 ▶).
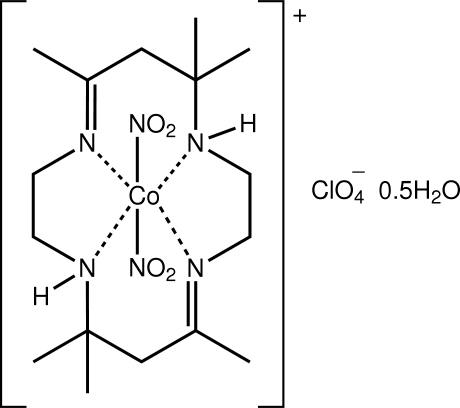


## Experimental

### 

#### Crystal data

[Co(NO_2_)_2_(C_16_H_32_N_4_)]ClO_4_·0.5H_2_O
*M* *_r_* = 539.87
Monoclinic, 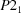

*a* = 15.7241 (5) Å
*b* = 6.8989 (2) Å
*c* = 20.6600 (8) Å
β = 97.196 (3)°
*V* = 2223.52 (13) Å^3^
*Z* = 4
Mo *K*α radiation
μ = 0.95 mm^−1^
*T* = 100 K
0.35 × 0.35 × 0.35 mm

#### Data collection

Agilent Technologies SuperNova Dual diffractometer with Atlas detector
Absorption correction: multi-scan (*CrysAlis PRO*; Agilent, 2010 ▶) *T*_min_ = 0.794, *T*_max_ = 1.000
16801 measured reflections
13634 independent reflections
12641 reflections with *I* > 2σ(*I*)
*R*_int_ = 0.095

#### Refinement

*R*[*F* ^2^ > 2σ(*F* ^2^)] = 0.043
*wR*(*F* ^2^) = 0.135
*S* = 1.07
13634 reflections
599 parameters
67 restraints
H-atom parameters constrained
Δρ_max_ = 0.46 e Å^−3^
Δρ_min_ = −0.73 e Å^−3^
Absolute structure: Flack (1983 ▶); 8210 Friedel pairs
Flack parameter: 0.509 (17)

### 

Data collection: *CrysAlis PRO* (Agilent, 2010 ▶); cell refinement: *CrysAlis PRO*; data reduction: *CrysAlis PRO*; program(s) used to solve structure: *SHELXS97* (Sheldrick, 2008 ▶); program(s) used to refine structure: *SHELXL97* (Sheldrick, 2008 ▶); molecular graphics: *ORTEP-3* (Farrugia, 1997 ▶) and *DIAMOND* (Brandenburg, 2006 ▶); software used to prepare material for publication: *publCIF* (Westrip, 2010 ▶).

## Supplementary Material

Crystal structure: contains datablock(s) global, I. DOI: 10.1107/S1600536811042784/hb6448sup1.cif

Structure factors: contains datablock(s) I. DOI: 10.1107/S1600536811042784/hb6448Isup2.hkl

Additional supplementary materials:  crystallographic information; 3D view; checkCIF report

## Figures and Tables

**Table 1:** Selected bond lengths (Å)

Co1—N1	1.933 (4)
Co1—N2	1.977 (4)
Co1—N3	1.930 (4)
Co1—N4	1.982 (3)
Co1—N5	1.992 (4)
Co1—N6	1.926 (4)
Co2—N7	1.937 (4)
Co2—N8	1.959 (4)
Co2—N9	1.933 (4)
Co2—N10	1.970 (3)
Co2—N11	1.937 (4)
Co2—N12	2.009 (4)

**Table 2:** Hydrogen-bond geometry (Å, °)

*D*—H⋯*A*	*D*—H	H⋯*A*	*D*⋯*A*	*D*—H⋯*A*
N2—H2⋯O2	0.88	2.02	2.718 (5)	135
N4—H4⋯O1	0.88	2.02	2.718 (5)	135
N8—H8⋯O7	0.88	2.04	2.744 (5)	136
N10—H10⋯O8	0.88	2.03	2.732 (5)	136
O1*W*—H1*W*⋯O9	0.85	2.24	2.907 (7)	135
O1*W*—H2*W*⋯O10^i^	0.85	2.34	2.988 (7)	134
C1—H1*A*⋯O6	0.99	2.46	3.428 (6)	166
C6—H6*B*⋯O13^ii^	0.99	2.45	3.426 (6)	169
C8—H8*A*⋯O2^i^	0.98	2.53	3.352 (6)	141
C8—H8*C*⋯O7^iii^	0.98	2.58	3.549 (6)	169
C9—H9*A*⋯O1^i^	0.99	2.53	3.441 (6)	153
C10—H10*A*⋯O1*W*^i^	0.99	2.59	3.115 (8)	114
C10—H10*A*⋯O10^i^	0.99	2.59	3.256 (6)	125
C16—H16*A*⋯O3^iv^	0.98	2.46	3.384 (6)	157
C16—H16*B*⋯O6	0.98	2.53	3.329 (6)	138
C17—H17*B*⋯O7^i^	0.99	2.59	3.426 (6)	142
C18—H18*B*⋯O10^v^	0.99	2.53	3.230 (6)	128
C20—H20*A*⋯O12^vi^	0.98	2.57	3.424 (7)	145
C21—H21*B*⋯O12^vii^	0.98	2.57	3.535 (6)	167
C24—H24*A*⋯O6^iv^	0.98	2.53	3.401 (6)	148
C25—H25*B*⋯O3^iv^	0.99	2.47	3.421 (6)	161
C26—H26*B*⋯O15	0.99	2.37	3.262 (6)	149
C28—H28*B*⋯O14^i^	0.98	2.56	3.264 (6)	129
C32—H32*B*⋯O14^viii^	0.98	2.45	3.384 (6)	159

Symmetry codes: (i) 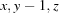; (ii) 
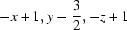
; (iii) 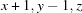; (iv) 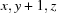; (v) 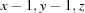; (vi) 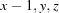; (vii) 
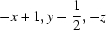
; (viii) 
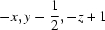
.

**Fig. 1.**
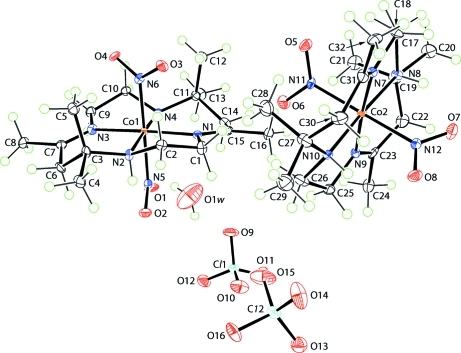
The molecular structures of the components comprising the asymmetric unit of (I) showing displacement ellipsoids at the 50% probability level.

**Fig. 2.**
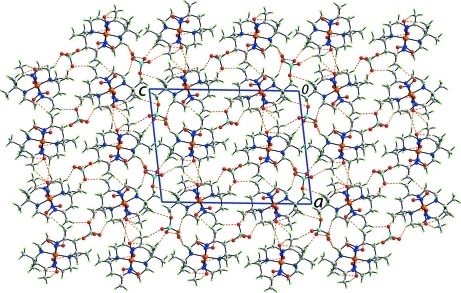
A view in projection down the b axis of the unit-cell contents of (I) showing the alternating layers of complex cations and anions stacking along the c axis. The O—H···O (largely obscured) and C—H···O interactions are shown as blue and orange dashed lines. respectively.

## supplementary crystallographic information

### Comment

The title complex, (I), was investigated as a part of continuing studies into the biocidal potential and structural properties of macrocyclic metal complexes (Roy *et al.* 2006; Hazari *et al.*, 2008).

The asymmetric unit of (I) comprises two independent complex cations, two perchlorate anions and a water molecule of crystallization, Fig. 1. The Co^III^ atom is coordinated by the four nitrogen atoms of the macrocycle and two nitrogen atoms derived from the nitrite anions. The resulting N_6_ coordination geometry is based on an octahedron. The Co—N distances span a relatively narrow range, *i.e*. 1.926 (4) to 2.009 (4) Å, Table 1. The shortest and longest Co—N distances involve Co—N(nitrite) bonds, and it is notable that one Co—N(nitrite) bond is systematically longer than the other in each complex cation. This feature of the bonding is readily explained by the presence of intramolecular N—H···O(nitrite) hydrogen bonds, Table 2. In each complex cation, one nitrite group (*i.e*. N5- and N12-) is orientated to optimize the formation of N—H···O hydrogen bonds as each nitrite-O atom is aligned with an amine-H atom, which lie to the same side of the CoN_4_ plane. The relative lengthening of the Co—N5, N12 bonds compensates for the weakening of the respective N—O bonds. Only small differences in the molecular structures are manifested: the r.m.s. deviations in bond distances and angles are 0.0132 Å and 0.928°, respectively (Spek, 2009). In terms of conformations, a small difference in the relative orientations in the nitrite anions is noted. Thus, for the Co1 complex, the dihedral angle formed between these is 55.1 (4)° which compares to 62.6 (4)° in the Co2 complex.

In the crystal packing, the water molecule of solvation bridges two Cl1-perchlorate anions, forming donor O—H···O interactions, Table 2. The result of this hydrogen bonding is the formation of supramolecular chains along the *b* axis comprising alternating water molecules and Cl-perchlorate anions. The water-O atom exists in a pocket of C-bound hydrogen atoms and the closest C—H···O(water) contact is weak, Table 2. The remaining intermolecular interactions are of the type C—H···O, Table 2, which serve to consolidate the three-dimensional architecture, Fig. 2. Globally, the crystal structure comprises layers of complex molecules in the *ab* plane comprising alternating rows of Co1- and Co2-containing complex molecules. Interspersing layers of complex cations are alternating layers comprising the Cl1-perchlorate anions and water molecules or Cl2-perchlorate anions.

### Experimental

The macrocycle, *L*, (Curtis & Hay, 1966) and precursor complex (Bembi *et al.*, 1984) were prepared as described in the literature. Sodium nitrite (0.069 g, 1.0 mmol) and *trans*-[CoLCl_2_](C1O_4_) (0.252 g, 0.5 mmol) were suspended in methanol (20 ml). After heating the mixture on a water-bath for 15 minutes, the yellow solution was filtered while hot. The filtrate was concentrated on a water bath until crystallization commenced. After cooling, the yellow product *trans*-[CoL(NO_2_)_2_](ClO_4_) was filtered off, washed with dry ethanol, followed by diethyl ether and finally dried *in vacuo. M*.pt:. 495–497 K. Yield 76.2%. FT—IR (KBr, cm^-1^): 3120 (s, νN-H); 1649 (s, νC═N); 1079, 622 (*versus*, νClO_4_); 519 (s, νCo-N); 117 5(m, νC—C); 2920 (vw, νCH); 1380 (s, νCH_3_); 1396 (*versus*, ν*_asym_*NO_2_); 1310 (*versus*, ν*_sym_*NO_2_).  Orange blocks of the title hemihydrate were prepared by slow evaporation of its acetonitrile and ethanol (1:1) mixture.

### Refinement

The N– and C-bound H-atoms were placed in calculated positions (N—H = 0.88 Å and C—H = 0.98–0.99 Å) and were included in the refinement in the riding model approximation, with *U*_iso_(H) set to 1.2*U*_equiv_(N,*C*). The water-H atoms were located from a difference map, fixed in these positions are refined with *U*_iso_(H) = 1.5*U*_equiv_(O). Several of the atoms (*i.e*. N4, N5, N7, C2, C5, C7, C9, C10, C11, C12 and C29) exhibited large displacement parameters and these were refined to be nearly isotropic with the ISOR command in *SHELXL97* (Sheldrick, 2008). The crystal is a non-merohedral, racemic twin. The two twin domains were separated by the diffractometer software and the intensities were integrated simultaneously; the two domains were scaled separately. The proportion of the twin domains refined to 0.644 (1): 0.356 (1). The Flack parameter refined to 0.509 (17) from 8210 Friedel pairs, *i.e.* the crystal is a racemic twin.

### Crystal data

**Table:**

[Co(NO_2_)_2_(C_16_H_32_N_4_)]ClO_4_·0.5H_2_O	*F*(000) = 1132
*M**_r_* = 539.87	*D*_x_ = 1.613 Mg m^−^^3^
Monoclinic, *P*2_1_	Mo *K*α radiation, λ = 0.71073 Å
Hall symbol: P 2yb	Cell parameters from 7862 reflections
*a* = 15.7241 (5) Å	θ = 2.2–27.5°
*b* = 6.8989 (2) Å	µ = 0.95 mm^−^^1^
*c* = 20.6600 (8) Å	*T* = 100 K
β = 97.196 (3)°	Block, orange
*V* = 2223.52 (13) Å^3^	0.35 × 0.35 × 0.35 mm
*Z* = 4	

### Data collection

**Table:**

Agilent Technologies SuperNova Dual diffractometer with Atlas detector	13634 independent reflections
Radiation source: SuperNova (Mo) X-ray Source	12641 reflections with *I* > 2σ(*I*)
Mirror	*R*_int_ = 0.095
Detector resolution: 10.4041 pixels mm^-1^	θ_max_ = 27.6°, θ_min_ = 2.2°
ω scan	*h* = −20→20
Absorption correction: multi-scan (*CrysAlis PRO*; Agilent, 2010)	*k* = −8→8
*T*_min_ = 0.794, *T*_max_ = 1.000	*l* = −25→26
16801 measured reflections	

### Refinement

**Table:**

Refinement on *F*^2^	Secondary atom site location: difference Fourier map
Least-squares matrix: full	Hydrogen site location: inferred from neighbouring sites
*R*[*F*^2^ > 2σ(*F*^2^)] = 0.043	H-atom parameters constrained
*wR*(*F*^2^) = 0.135	*w* = 1/[σ^2^(*F*_o_^2^) + (0.0839*P*)^2^] where *P* = (*F*_o_^2^ + 2*F*_c_^2^)/3
*S* = 1.07	(Δ/σ)_max_ = 0.001
13634 reflections	Δρ_max_ = 0.46 e Å^−^^3^
599 parameters	Δρ_min_ = −0.73 e Å^−^^3^
67 restraints	Absolute structure: Flack (1983); 8210 Friedel pairs
Primary atom site location: structure-invariant direct methods	Flack parameter: 0.509 (17)

### Special details

**Table:**

Geometry. All e.s.d.'s (except the e.s.d. in the dihedral angle between two l.s. planes) are estimated using the full covariance matrix. The cell e.s.d.'s are taken into account individually in the estimation of e.s.d.'s in distances, angles and torsion angles; correlations between e.s.d.'s in cell parameters are only used when they are defined by crystal symmetry. An approximate (isotropic) treatment of cell e.s.d.'s is used for estimating e.s.d.'s involving l.s. planes.
Refinement. Refinement of *F*^2^ against ALL reflections. The weighted *R*-factor *wR* and goodness of fit *S* are based on *F*^2^, conventional *R*-factors *R* are based on *F*, with *F* set to zero for negative *F*^2^. The threshold expression of *F*^2^ > σ(*F*^2^) is used only for calculating *R*-factors(gt) *etc*. and is not relevant to the choice of reflections for refinement. *R*-factors based on *F*^2^ are statistically about twice as large as those based on *F*, and *R*- factors based on ALL data will be even larger.

### Fractional atomic coordinates and isotropic or equivalent isotropic displacement parameters (Å^2^)

**Table:**

	*x*	*y*	*z*	*U*_iso_*/*U*_eq_	
Co1	0.51998 (3)	0.49995 (7)	0.24819 (3)	0.00692 (12)	
Co2	0.01422 (3)	0.97924 (8)	0.25404 (3)	0.00800 (13)	
Cl1	0.77354 (7)	1.47781 (19)	0.03181 (5)	0.0185 (2)	
Cl2	0.27764 (7)	1.43839 (17)	0.49275 (6)	0.0174 (2)	
O1	0.6409 (2)	0.7731 (5)	0.21375 (16)	0.0163 (7)	
O2	0.6015 (2)	0.8328 (5)	0.30666 (16)	0.0161 (7)	
O3	0.3701 (2)	0.2859 (5)	0.24567 (17)	0.0156 (7)	
O4	0.4778 (2)	0.1115 (4)	0.22963 (17)	0.0142 (7)	
O5	0.0422 (2)	0.5864 (4)	0.23665 (17)	0.0169 (7)	
O6	0.1593 (2)	0.7499 (5)	0.25060 (18)	0.0185 (7)	
O7	−0.1090 (2)	1.2745 (5)	0.21914 (17)	0.0196 (8)	
O8	−0.0481 (2)	1.3102 (5)	0.31662 (17)	0.0181 (7)	
O9	0.7334 (3)	1.2986 (6)	0.00821 (19)	0.0355 (10)	
O10	0.7360 (2)	1.5462 (6)	0.08771 (19)	0.0340 (10)	
O11	0.7609 (3)	1.6216 (6)	−0.0189 (2)	0.0392 (11)	
O12	0.8630 (2)	1.4462 (6)	0.04931 (19)	0.0316 (9)	
O13	0.2927 (2)	1.6370 (5)	0.47582 (18)	0.0265 (9)	
O14	0.1900 (2)	1.4119 (6)	0.5032 (2)	0.0378 (11)	
O15	0.2988 (3)	1.3121 (6)	0.44121 (19)	0.0389 (11)	
O16	0.3315 (2)	1.3903 (6)	0.55179 (17)	0.0293 (9)	
O1W	0.6926 (4)	0.9679 (9)	0.0859 (3)	0.0860 (19)	
H1w	0.7183	1.0764	0.0853	0.129*	
H2w	0.7260	0.8822	0.0738	0.129*	
N1	0.4211 (2)	0.6657 (5)	0.22789 (19)	0.0092 (8)	
N2	0.5008 (2)	0.5382 (5)	0.34007 (17)	0.0093 (8)	
H2	0.5266	0.6489	0.3507	0.011*	
N3	0.6214 (2)	0.3431 (6)	0.26877 (19)	0.0114 (8)	
N4	0.5454 (2)	0.4761 (5)	0.15697 (15)	0.0072 (6)	
H4	0.5848	0.5653	0.1541	0.009*	
N5	0.5956 (2)	0.7325 (5)	0.25722 (19)	0.0106 (8)	
N6	0.4479 (2)	0.2733 (6)	0.23987 (18)	0.0106 (8)	
N7	−0.0814 (2)	0.8350 (6)	0.28043 (19)	0.0103 (8)	
N8	−0.0454 (2)	0.9498 (6)	0.16546 (17)	0.0097 (8)	
H8	−0.0822	1.0463	0.1622	0.012*	
N9	0.1049 (2)	1.1387 (5)	0.22762 (19)	0.0116 (8)	
N10	0.0702 (2)	1.0221 (5)	0.34365 (16)	0.0084 (7)	
H10	0.0492	1.1344	0.3541	0.010*	
N11	0.0792 (2)	0.7439 (5)	0.24578 (18)	0.0096 (8)	
N12	−0.0550 (2)	1.2198 (5)	0.26383 (19)	0.0110 (8)	
C1	0.3777 (3)	0.7146 (7)	0.2855 (2)	0.0137 (10)	
H1A	0.3149	0.7007	0.2742	0.016*	
H1B	0.3900	0.8511	0.2982	0.016*	
C2	0.4080 (3)	0.5835 (7)	0.3419 (2)	0.0125 (9)	
H2A	0.4004	0.6481	0.3835	0.015*	
H2B	0.3741	0.4623	0.3387	0.015*	
C3	0.5399 (3)	0.4014 (6)	0.3918 (2)	0.0116 (9)	
C4	0.5304 (3)	0.4886 (8)	0.4587 (2)	0.0160 (9)	
H4A	0.4714	0.4703	0.4682	0.024*	
H4B	0.5435	0.6274	0.4584	0.024*	
H4C	0.5700	0.4239	0.4922	0.024*	
C5	0.5010 (3)	0.1996 (7)	0.3867 (2)	0.0159 (10)	
H5A	0.5115	0.1402	0.3453	0.024*	
H5B	0.4391	0.2085	0.3884	0.024*	
H5C	0.5273	0.1199	0.4231	0.024*	
C6	0.6364 (3)	0.3948 (7)	0.3862 (2)	0.0137 (10)	
H6A	0.6589	0.5287	0.3912	0.016*	
H6B	0.6646	0.3184	0.4234	0.016*	
C7	0.6635 (3)	0.3122 (6)	0.3249 (2)	0.0110 (9)	
C8	0.7444 (3)	0.1935 (7)	0.3337 (2)	0.0146 (10)	
H8A	0.7300	0.0560	0.3274	0.022*	
H8B	0.7740	0.2131	0.3778	0.022*	
H8C	0.7819	0.2339	0.3017	0.022*	
C9	0.6566 (3)	0.2704 (7)	0.2100 (2)	0.0131 (9)	
H9A	0.6727	0.1324	0.2164	0.016*	
H9B	0.7090	0.3443	0.2039	0.016*	
C10	0.5923 (3)	0.2903 (6)	0.1499 (2)	0.0114 (9)	
H10A	0.6217	0.2935	0.1103	0.014*	
H10B	0.5519	0.1796	0.1464	0.014*	
C11	0.4776 (3)	0.5232 (7)	0.1011 (2)	0.0111 (9)	
C12	0.4050 (3)	0.3767 (7)	0.0921 (2)	0.0151 (10)	
H12A	0.3765	0.3725	0.1317	0.023*	
H12B	0.4282	0.2482	0.0841	0.023*	
H12C	0.3636	0.4147	0.0549	0.023*	
C13	0.5202 (3)	0.5415 (7)	0.0388 (2)	0.0170 (10)	
H13A	0.5697	0.6286	0.0467	0.025*	
H13B	0.4789	0.5943	0.0037	0.025*	
H13C	0.5393	0.4135	0.0261	0.025*	
C14	0.4424 (3)	0.7242 (7)	0.1149 (2)	0.0129 (9)	
H14A	0.4912	0.8158	0.1202	0.016*	
H14B	0.4038	0.7652	0.0757	0.016*	
C15	0.3943 (3)	0.7472 (6)	0.1732 (2)	0.0108 (9)	
C16	0.3156 (3)	0.8708 (7)	0.1635 (2)	0.0163 (10)	
H16A	0.3206	0.9753	0.1959	0.024*	
H16B	0.2652	0.7914	0.1688	0.024*	
H16C	0.3093	0.9267	0.1196	0.024*	
C17	−0.1415 (3)	0.7650 (7)	0.2246 (2)	0.0153 (10)	
H17A	−0.1943	0.8447	0.2205	0.018*	
H17B	−0.1577	0.6292	0.2325	0.018*	
C18	−0.1013 (3)	0.7762 (7)	0.1619 (2)	0.0145 (10)	
H18A	−0.0672	0.6580	0.1566	0.017*	
H18B	−0.1465	0.7865	0.1241	0.017*	
C19	0.0020 (3)	0.9785 (8)	0.10736 (19)	0.0147 (9)	
C20	−0.0625 (3)	0.9806 (8)	0.0452 (2)	0.0206 (10)	
H20A	−0.1062	1.0798	0.0492	0.031*	
H20B	−0.0900	0.8533	0.0391	0.031*	
H20C	−0.0326	1.0101	0.0075	0.031*	
C21	0.0677 (3)	0.8187 (7)	0.1013 (2)	0.0188 (11)	
H21A	0.1125	0.8244	0.1388	0.028*	
H21B	0.0935	0.8369	0.0610	0.028*	
H21C	0.0393	0.6921	0.1004	0.028*	
C22	0.0456 (3)	1.1759 (7)	0.1147 (2)	0.0179 (11)	
H22A	0.0731	1.1996	0.0748	0.022*	
H22B	0.0002	1.2750	0.1156	0.022*	
C23	0.1112 (3)	1.2107 (6)	0.1720 (2)	0.0138 (10)	
C24	0.1818 (3)	1.3476 (7)	0.1591 (2)	0.0173 (10)	
H24A	0.1943	1.4360	0.1962	0.026*	
H24B	0.1635	1.4227	0.1196	0.026*	
H24C	0.2334	1.2734	0.1532	0.026*	
C25	0.1684 (3)	1.1964 (7)	0.2841 (2)	0.0139 (10)	
H25A	0.1577	1.3321	0.2963	0.017*	
H25B	0.2270	1.1889	0.2714	0.017*	
C26	0.1619 (3)	1.0651 (7)	0.3419 (2)	0.0190 (10)	
H26A	0.1941	0.9436	0.3373	0.023*	
H26B	0.1863	1.1301	0.3828	0.023*	
C27	0.0498 (3)	0.8906 (7)	0.3978 (2)	0.0181 (10)	
C28	0.0840 (3)	0.6852 (7)	0.3920 (2)	0.0217 (11)	
H28A	0.0516	0.6203	0.3545	0.033*	
H28B	0.0776	0.6126	0.4318	0.033*	
H28C	0.1448	0.6906	0.3858	0.033*	
C29	0.0903 (3)	0.9747 (9)	0.4635 (2)	0.0252 (10)	
H29A	0.0751	1.1120	0.4659	0.038*	
H29B	0.1528	0.9618	0.4671	0.038*	
H29C	0.0689	0.9039	0.4992	0.038*	
C30	−0.0460 (3)	0.8849 (7)	0.3975 (2)	0.0186 (11)	
H30A	−0.0656	1.0187	0.4046	0.022*	
H30B	−0.0583	0.8069	0.4354	0.022*	
C31	−0.1002 (3)	0.8057 (7)	0.3377 (2)	0.0148 (10)	
C32	−0.1776 (3)	0.6968 (6)	0.3511 (2)	0.0156 (10)	
H32A	−0.2238	0.7176	0.3153	0.023*	
H32B	−0.1959	0.7426	0.3921	0.023*	
H32C	−0.1641	0.5582	0.3547	0.023*	

### Atomic displacement parameters (Å^2^)

**Table:**

	*U*^11^	*U*^22^	*U*^33^	*U*^12^	*U*^13^	*U*^23^
Co1	0.0060 (2)	0.0075 (3)	0.0074 (2)	0.0003 (3)	0.0012 (2)	0.0003 (2)
Co2	0.0063 (2)	0.0083 (3)	0.0096 (3)	−0.0010 (3)	0.0015 (2)	−0.0011 (3)
Cl1	0.0191 (5)	0.0164 (5)	0.0189 (5)	−0.0018 (5)	−0.0013 (4)	−0.0017 (5)
Cl2	0.0161 (5)	0.0208 (6)	0.0149 (5)	−0.0034 (4)	0.0006 (4)	0.0002 (5)
O1	0.0209 (17)	0.0118 (16)	0.0179 (17)	−0.0089 (14)	0.0093 (15)	−0.0027 (14)
O2	0.0204 (18)	0.0124 (15)	0.0152 (17)	−0.0036 (14)	0.0009 (14)	−0.0029 (14)
O3	0.0099 (15)	0.0192 (17)	0.0176 (18)	−0.0047 (13)	0.0019 (14)	0.0026 (15)
O4	0.0169 (16)	0.0059 (15)	0.0199 (18)	0.0003 (12)	0.0023 (14)	−0.0005 (13)
O5	0.0166 (16)	0.0122 (16)	0.0225 (19)	0.0027 (13)	0.0053 (14)	−0.0021 (14)
O6	0.0104 (16)	0.0210 (17)	0.0244 (19)	0.0017 (13)	0.0033 (15)	−0.0027 (17)
O7	0.0236 (19)	0.0171 (17)	0.0181 (18)	0.0082 (15)	0.0023 (16)	0.0026 (15)
O8	0.0169 (17)	0.0145 (17)	0.0237 (19)	0.0000 (14)	0.0054 (14)	−0.0065 (15)
O9	0.048 (3)	0.021 (2)	0.034 (2)	−0.0139 (19)	−0.011 (2)	−0.0063 (18)
O10	0.0256 (19)	0.044 (2)	0.035 (2)	−0.0093 (18)	0.0116 (17)	−0.0127 (19)
O11	0.052 (3)	0.030 (2)	0.034 (2)	0.004 (2)	−0.001 (2)	0.010 (2)
O12	0.0192 (17)	0.034 (2)	0.040 (2)	0.0037 (17)	−0.0033 (17)	−0.0135 (19)
O13	0.029 (2)	0.025 (2)	0.025 (2)	−0.0018 (17)	−0.0001 (17)	0.0015 (17)
O14	0.0163 (18)	0.059 (3)	0.039 (2)	−0.0011 (18)	0.0060 (18)	0.026 (2)
O15	0.054 (3)	0.046 (2)	0.019 (2)	−0.017 (2)	0.010 (2)	−0.0153 (19)
O16	0.026 (2)	0.043 (2)	0.0160 (19)	0.0018 (17)	−0.0078 (16)	0.0028 (17)
O1W	0.124 (5)	0.053 (3)	0.091 (4)	0.016 (4)	0.054 (4)	0.002 (4)
N1	0.0100 (19)	0.0081 (18)	0.0096 (19)	−0.0002 (15)	0.0009 (15)	−0.0024 (15)
N2	0.0088 (17)	0.0118 (19)	0.0079 (18)	0.0022 (14)	0.0038 (13)	0.0020 (15)
N3	0.0070 (17)	0.0123 (18)	0.016 (2)	−0.0014 (15)	0.0054 (16)	0.0032 (16)
N4	0.0084 (9)	0.0065 (10)	0.0071 (9)	0.0003 (9)	0.0026 (8)	−0.0005 (9)
N5	0.0098 (11)	0.0109 (11)	0.0107 (11)	0.0016 (8)	0.0002 (8)	0.0009 (9)
N6	0.015 (2)	0.0103 (19)	0.0077 (19)	0.0022 (15)	0.0045 (16)	0.0002 (15)
N7	0.0086 (11)	0.0098 (11)	0.0122 (11)	0.0001 (8)	0.0003 (8)	0.0001 (9)
N8	0.0098 (16)	0.0134 (19)	0.0067 (17)	−0.0021 (15)	0.0039 (13)	−0.0011 (15)
N9	0.0057 (17)	0.0112 (19)	0.018 (2)	0.0007 (15)	0.0018 (16)	−0.0020 (17)
N10	0.0091 (16)	0.0090 (18)	0.0075 (16)	0.0014 (14)	0.0019 (13)	−0.0038 (14)
N11	0.0100 (17)	0.0086 (18)	0.0108 (19)	0.0023 (15)	0.0033 (15)	0.0036 (16)
N12	0.0078 (17)	0.010 (2)	0.016 (2)	−0.0010 (15)	0.0044 (15)	0.0029 (17)
C1	0.011 (2)	0.019 (2)	0.012 (2)	0.0053 (19)	0.0061 (18)	0.001 (2)
C2	0.0121 (12)	0.0134 (12)	0.0123 (12)	0.0004 (9)	0.0032 (9)	−0.0011 (9)
C3	0.015 (2)	0.015 (2)	0.005 (2)	0.0032 (18)	0.0001 (17)	0.0013 (17)
C4	0.019 (2)	0.019 (2)	0.010 (2)	−0.002 (2)	−0.0004 (16)	0.003 (2)
C5	0.0161 (13)	0.0170 (13)	0.0143 (13)	0.0001 (9)	0.0013 (9)	0.0007 (9)
C6	0.013 (2)	0.017 (2)	0.010 (2)	−0.0035 (18)	−0.0040 (18)	0.0019 (19)
C7	0.0107 (12)	0.0106 (12)	0.0119 (12)	−0.0017 (9)	0.0023 (9)	0.0004 (9)
C8	0.009 (2)	0.013 (2)	0.022 (3)	0.0033 (18)	0.0032 (19)	−0.002 (2)
C9	0.0130 (12)	0.0132 (12)	0.0134 (12)	0.0024 (9)	0.0030 (9)	−0.0009 (9)
C10	0.0120 (12)	0.0111 (12)	0.0113 (12)	0.0004 (9)	0.0027 (9)	−0.0015 (9)
C11	0.0116 (12)	0.0121 (12)	0.0096 (12)	−0.0003 (9)	0.0014 (9)	0.0013 (9)
C12	0.0148 (12)	0.0168 (12)	0.0138 (12)	−0.0014 (9)	0.0028 (9)	0.0006 (9)
C13	0.021 (2)	0.022 (2)	0.008 (2)	0.000 (2)	0.0014 (18)	0.0008 (19)
C14	0.010 (2)	0.019 (2)	0.010 (2)	−0.0004 (19)	0.0037 (18)	−0.0011 (19)
C15	0.011 (2)	0.009 (2)	0.012 (2)	0.0004 (18)	0.0019 (17)	−0.0016 (17)
C16	0.017 (2)	0.014 (2)	0.018 (3)	0.0050 (19)	0.002 (2)	0.001 (2)
C17	0.010 (2)	0.014 (2)	0.022 (3)	−0.0051 (18)	0.0009 (19)	−0.001 (2)
C18	0.010 (2)	0.017 (2)	0.015 (2)	−0.0038 (19)	−0.0024 (18)	−0.006 (2)
C19	0.018 (2)	0.021 (2)	0.0061 (18)	0.002 (2)	0.0058 (16)	−0.004 (2)
C20	0.029 (2)	0.023 (2)	0.009 (2)	−0.002 (3)	0.0009 (18)	0.000 (2)
C21	0.023 (3)	0.024 (3)	0.011 (2)	0.003 (2)	0.009 (2)	−0.005 (2)
C22	0.020 (3)	0.016 (2)	0.018 (3)	−0.001 (2)	0.006 (2)	0.007 (2)
C23	0.013 (2)	0.006 (2)	0.024 (3)	0.0030 (17)	0.0068 (19)	0.0012 (19)
C24	0.016 (2)	0.018 (2)	0.020 (2)	0.002 (2)	0.007 (2)	0.007 (2)
C25	0.010 (2)	0.011 (2)	0.020 (2)	−0.0032 (18)	0.0025 (19)	0.001 (2)
C26	0.014 (2)	0.024 (3)	0.018 (2)	−0.005 (2)	−0.0005 (19)	−0.006 (2)
C27	0.020 (2)	0.022 (2)	0.012 (2)	−0.008 (2)	−0.001 (2)	0.006 (2)
C28	0.022 (3)	0.023 (3)	0.019 (3)	0.001 (2)	−0.001 (2)	0.004 (2)
C29	0.0260 (13)	0.0268 (13)	0.0225 (13)	−0.0002 (10)	0.0021 (9)	−0.0010 (10)
C30	0.026 (3)	0.017 (2)	0.012 (2)	−0.003 (2)	0.003 (2)	−0.001 (2)
C31	0.008 (2)	0.014 (2)	0.023 (3)	−0.0001 (18)	0.0049 (19)	0.005 (2)
C32	0.013 (2)	0.010 (2)	0.025 (3)	−0.0040 (18)	0.008 (2)	−0.001 (2)

### Geometric parameters (Å, °)

**Table:**

Co1—N1	1.933 (4)	C7—C8	1.505 (6)
Co1—N2	1.977 (4)	C8—H8A	0.9800
Co1—N3	1.930 (4)	C8—H8B	0.9800
Co1—N4	1.982 (3)	C8—H8C	0.9800
Co1—N5	1.992 (4)	C9—C10	1.506 (6)
Co1—N6	1.926 (4)	C9—H9A	0.9900
Co2—N7	1.937 (4)	C9—H9B	0.9900
Co2—N8	1.959 (4)	C10—H10A	0.9900
Co2—N9	1.933 (4)	C10—H10B	0.9900
Co2—N10	1.970 (3)	C11—C12	1.518 (6)
Co2—N11	1.937 (4)	C11—C13	1.529 (6)
Co2—N12	2.009 (4)	C11—C14	1.533 (6)
Cl1—O12	1.425 (4)	C12—H12A	0.9800
Cl1—O11	1.438 (4)	C12—H12B	0.9800
Cl1—O10	1.440 (4)	C12—H12C	0.9800
Cl1—O9	1.445 (4)	C13—H13A	0.9800
Cl2—O14	1.433 (4)	C13—H13B	0.9800
Cl2—O16	1.434 (4)	C13—H13C	0.9800
Cl2—O13	1.441 (4)	C14—C15	1.508 (6)
Cl2—O15	1.446 (4)	C14—H14A	0.9900
O1—N5	1.246 (5)	C14—H14B	0.9900
O2—N5	1.227 (5)	C15—C16	1.496 (6)
O3—N6	1.247 (5)	C16—H16A	0.9800
O4—N6	1.239 (5)	C16—H16B	0.9800
O5—N11	1.236 (5)	C16—H16C	0.9800
O6—N11	1.251 (5)	C17—C18	1.512 (6)
O7—N12	1.232 (5)	C17—H17A	0.9900
O8—N12	1.250 (5)	C17—H17B	0.9900
O1W—H1w	0.8517	C18—H18A	0.9900
O1W—H2w	0.8497	C18—H18B	0.9900
N1—C15	1.286 (6)	C19—C22	1.524 (7)
N1—C1	1.484 (5)	C19—C21	1.526 (7)
N2—C2	1.497 (5)	C19—C20	1.534 (6)
N2—C3	1.499 (5)	C20—H20A	0.9800
N2—H2	0.8800	C20—H20B	0.9800
N3—C7	1.279 (6)	C20—H20C	0.9800
N3—C9	1.483 (6)	C21—H21A	0.9800
N4—C10	1.495 (5)	C21—H21B	0.9800
N4—C11	1.505 (5)	C21—H21C	0.9800
N4—H4	0.8800	C22—C23	1.489 (7)
N7—C31	1.272 (6)	C22—H22A	0.9900
N7—C17	1.478 (6)	C22—H22B	0.9900
N8—C18	1.483 (6)	C23—C24	1.506 (6)
N8—C19	1.503 (5)	C24—H24A	0.9800
N8—H8	0.8800	C24—H24B	0.9800
N9—C23	1.267 (6)	C24—H24C	0.9800
N9—C25	1.491 (6)	C25—C26	1.513 (7)
N10—C26	1.477 (5)	C25—H25A	0.9900
N10—C27	1.506 (6)	C25—H25B	0.9900
N10—H10	0.8800	C26—H26A	0.9900
C1—C2	1.504 (6)	C26—H26B	0.9900
C1—H1A	0.9900	C27—C30	1.507 (7)
C1—H1B	0.9900	C27—C28	1.526 (7)
C2—H2A	0.9900	C27—C29	1.538 (7)
C2—H2B	0.9900	C28—H28A	0.9800
C3—C5	1.519 (6)	C28—H28B	0.9800
C3—C4	1.532 (6)	C28—H28C	0.9800
C3—C6	1.536 (6)	C29—H29A	0.9800
C4—H4A	0.9800	C29—H29B	0.9800
C4—H4B	0.9800	C29—H29C	0.9800
C4—H4C	0.9800	C30—C31	1.511 (7)
C5—H5A	0.9800	C30—H30A	0.9900
C5—H5B	0.9800	C30—H30B	0.9900
C5—H5C	0.9800	C31—C32	1.485 (6)
C6—C7	1.498 (6)	C32—H32A	0.9800
C6—H6A	0.9900	C32—H32B	0.9800
C6—H6B	0.9900	C32—H32C	0.9800
			
N6—Co1—N3	91.41 (16)	N3—C9—C10	111.3 (4)
N6—Co1—N1	90.74 (17)	N3—C9—H9A	109.4
N3—Co1—N1	177.85 (17)	C10—C9—H9A	109.4
N6—Co1—N2	92.01 (16)	N3—C9—H9B	109.4
N3—Co1—N2	94.84 (15)	C10—C9—H9B	109.4
N1—Co1—N2	85.07 (16)	H9A—C9—H9B	108.0
N6—Co1—N4	91.97 (15)	N4—C10—C9	106.6 (3)
N3—Co1—N4	84.47 (15)	N4—C10—H10A	110.4
N1—Co1—N4	95.47 (15)	C9—C10—H10A	110.4
N2—Co1—N4	175.98 (16)	N4—C10—H10B	110.4
N6—Co1—N5	179.36 (17)	C9—C10—H10B	110.4
N3—Co1—N5	87.96 (16)	H10A—C10—H10B	108.6
N1—Co1—N5	89.89 (16)	N4—C11—C12	113.5 (4)
N2—Co1—N5	87.92 (16)	N4—C11—C13	108.7 (3)
N4—Co1—N5	88.10 (15)	C12—C11—C13	110.7 (4)
N9—Co2—N11	92.08 (16)	N4—C11—C14	106.8 (3)
N9—Co2—N7	176.13 (16)	C12—C11—C14	110.0 (4)
N11—Co2—N7	91.77 (16)	C13—C11—C14	106.8 (4)
N9—Co2—N8	94.51 (16)	C11—C12—H12A	109.5
N11—Co2—N8	91.52 (16)	C11—C12—H12B	109.5
N7—Co2—N8	84.98 (16)	H12A—C12—H12B	109.5
N9—Co2—N10	85.49 (16)	C11—C12—H12C	109.5
N11—Co2—N10	91.71 (15)	H12A—C12—H12C	109.5
N7—Co2—N10	94.81 (15)	H12B—C12—H12C	109.5
N8—Co2—N10	176.77 (15)	C11—C13—H13A	109.5
N9—Co2—N12	89.23 (15)	C11—C13—H13B	109.5
N11—Co2—N12	178.64 (15)	H13A—C13—H13B	109.5
N7—Co2—N12	86.92 (16)	C11—C13—H13C	109.5
N8—Co2—N12	88.74 (16)	H13A—C13—H13C	109.5
N10—Co2—N12	88.03 (15)	H13B—C13—H13C	109.5
O12—Cl1—O11	109.4 (3)	C15—C14—C11	118.2 (4)
O12—Cl1—O10	109.7 (2)	C15—C14—H14A	107.8
O11—Cl1—O10	109.0 (3)	C11—C14—H14A	107.8
O12—Cl1—O9	109.3 (3)	C15—C14—H14B	107.8
O11—Cl1—O9	109.2 (3)	C11—C14—H14B	107.8
O10—Cl1—O9	110.2 (3)	H14A—C14—H14B	107.1
O14—Cl2—O16	108.6 (2)	N1—C15—C16	122.8 (4)
O14—Cl2—O13	110.4 (2)	N1—C15—C14	120.9 (4)
O16—Cl2—O13	109.2 (2)	C16—C15—C14	116.3 (4)
O14—Cl2—O15	110.4 (3)	C15—C16—H16A	109.5
O16—Cl2—O15	108.7 (3)	C15—C16—H16B	109.5
O13—Cl2—O15	109.5 (2)	H16A—C16—H16B	109.5
H1w—O1w—H2w	107.0	C15—C16—H16C	109.5
C15—N1—C1	118.2 (4)	H16A—C16—H16C	109.5
C15—N1—Co1	128.0 (3)	H16B—C16—H16C	109.5
C1—N1—Co1	113.5 (3)	N7—C17—C18	110.9 (4)
C2—N2—C3	115.4 (3)	N7—C17—H17A	109.5
C2—N2—Co1	108.5 (3)	C18—C17—H17A	109.5
C3—N2—Co1	120.2 (3)	N7—C17—H17B	109.5
C2—N2—H2	103.5	C18—C17—H17B	109.5
C3—N2—H2	103.5	H17A—C17—H17B	108.1
Co1—N2—H2	103.5	N8—C18—C17	107.9 (4)
C7—N3—C9	118.8 (4)	N8—C18—H18A	110.1
C7—N3—Co1	127.8 (3)	C17—C18—H18A	110.1
C9—N3—Co1	113.1 (3)	N8—C18—H18B	110.1
C10—N4—C11	114.9 (3)	C17—C18—H18B	110.1
C10—N4—Co1	108.9 (3)	H18A—C18—H18B	108.4
C11—N4—Co1	120.2 (2)	N8—C19—C22	107.5 (4)
C10—N4—H4	103.5	N8—C19—C21	112.2 (4)
C11—N4—H4	103.5	C22—C19—C21	110.7 (4)
Co1—N4—H4	103.5	N8—C19—C20	109.1 (3)
O2—N5—O1	118.9 (4)	C22—C19—C20	108.4 (4)
O2—N5—Co1	120.9 (3)	C21—C19—C20	108.8 (4)
O1—N5—Co1	120.1 (3)	C19—C20—H20A	109.5
O4—N6—O3	118.4 (4)	C19—C20—H20B	109.5
O4—N6—Co1	121.0 (3)	H20A—C20—H20B	109.5
O3—N6—Co1	120.5 (3)	C19—C20—H20C	109.5
C31—N7—C17	118.3 (4)	H20A—C20—H20C	109.5
C31—N7—Co2	128.5 (3)	H20B—C20—H20C	109.5
C17—N7—Co2	113.0 (3)	C19—C21—H21A	109.5
C18—N8—C19	114.7 (4)	C19—C21—H21B	109.5
C18—N8—Co2	110.0 (3)	H21A—C21—H21B	109.5
C19—N8—Co2	120.4 (3)	C19—C21—H21C	109.5
C18—N8—H8	103.1	H21A—C21—H21C	109.5
C19—N8—H8	103.1	H21B—C21—H21C	109.5
Co2—N8—H8	103.1	C23—C22—C19	118.7 (4)
C23—N9—C25	119.0 (4)	C23—C22—H22A	107.6
C23—N9—Co2	128.6 (3)	C19—C22—H22A	107.6
C25—N9—Co2	112.1 (3)	C23—C22—H22B	107.6
C26—N10—C27	116.0 (4)	C19—C22—H22B	107.6
C26—N10—Co2	109.3 (3)	H22A—C22—H22B	107.1
C27—N10—Co2	119.8 (3)	N9—C23—C22	122.2 (4)
C26—N10—H10	103.1	N9—C23—C24	123.3 (4)
C27—N10—H10	103.1	C22—C23—C24	114.4 (4)
Co2—N10—H10	103.1	C23—C24—H24A	109.5
O5—N11—O6	119.4 (4)	C23—C24—H24B	109.5
O5—N11—Co2	120.4 (3)	H24A—C24—H24B	109.5
O6—N11—Co2	120.1 (3)	C23—C24—H24C	109.5
O7—N12—O8	118.5 (4)	H24A—C24—H24C	109.5
O7—N12—Co2	120.6 (3)	H24B—C24—H24C	109.5
O8—N12—Co2	120.8 (3)	N9—C25—C26	110.7 (4)
N1—C1—C2	110.5 (4)	N9—C25—H25A	109.5
N1—C1—H1A	109.5	C26—C25—H25A	109.5
C2—C1—H1A	109.5	N9—C25—H25B	109.5
N1—C1—H1B	109.5	C26—C25—H25B	109.5
C2—C1—H1B	109.5	H25A—C25—H25B	108.1
H1A—C1—H1B	108.1	N10—C26—C25	107.6 (4)
N2—C2—C1	108.8 (3)	N10—C26—H26A	110.2
N2—C2—H2A	109.9	C25—C26—H26A	110.2
C1—C2—H2A	109.9	N10—C26—H26B	110.2
N2—C2—H2B	109.9	C25—C26—H26B	110.2
C1—C2—H2B	109.9	H26A—C26—H26B	108.5
H2A—C2—H2B	108.3	N10—C27—C30	108.6 (4)
N2—C3—C5	113.7 (4)	N10—C27—C28	113.0 (4)
N2—C3—C4	108.5 (4)	C30—C27—C28	109.7 (4)
C5—C3—C4	109.8 (4)	N10—C27—C29	109.0 (4)
N2—C3—C6	106.6 (3)	C30—C27—C29	108.5 (4)
C5—C3—C6	111.1 (4)	C28—C27—C29	108.0 (4)
C4—C3—C6	106.8 (4)	C27—C28—H28A	109.5
C3—C4—H4A	109.5	C27—C28—H28B	109.5
C3—C4—H4B	109.5	H28A—C28—H28B	109.5
H4A—C4—H4B	109.5	C27—C28—H28C	109.5
C3—C4—H4C	109.5	H28A—C28—H28C	109.5
H4A—C4—H4C	109.5	H28B—C28—H28C	109.5
H4B—C4—H4C	109.5	C27—C29—H29A	109.5
C3—C5—H5A	109.5	C27—C29—H29B	109.5
C3—C5—H5B	109.5	H29A—C29—H29B	109.5
H5A—C5—H5B	109.5	C27—C29—H29C	109.5
C3—C5—H5C	109.5	H29A—C29—H29C	109.5
H5A—C5—H5C	109.5	H29B—C29—H29C	109.5
H5B—C5—H5C	109.5	C27—C30—C31	118.3 (4)
C7—C6—C3	117.6 (4)	C27—C30—H30A	107.7
C7—C6—H6A	107.9	C31—C30—H30A	107.7
C3—C6—H6A	107.9	C27—C30—H30B	107.7
C7—C6—H6B	107.9	C31—C30—H30B	107.7
C3—C6—H6B	107.9	H30A—C30—H30B	107.1
H6A—C6—H6B	107.2	N7—C31—C32	123.0 (4)
N3—C7—C6	122.2 (4)	N7—C31—C30	122.0 (4)
N3—C7—C8	122.3 (4)	C32—C31—C30	115.0 (4)
C6—C7—C8	115.5 (4)	C31—C32—H32A	109.5
C7—C8—H8A	109.5	C31—C32—H32B	109.5
C7—C8—H8B	109.5	H32A—C32—H32B	109.5
H8A—C8—H8B	109.5	C31—C32—H32C	109.5
C7—C8—H8C	109.5	H32A—C32—H32C	109.5
H8A—C8—H8C	109.5	H32B—C32—H32C	109.5
H8B—C8—H8C	109.5		
			
N6—Co1—N1—C15	−100.5 (4)	N9—Co2—N12—O7	95.6 (3)
N2—Co1—N1—C15	167.5 (4)	N7—Co2—N12—O7	−84.0 (3)
N4—Co1—N1—C15	−8.5 (4)	N8—Co2—N12—O7	1.1 (3)
N5—Co1—N1—C15	79.6 (4)	N10—Co2—N12—O7	−178.9 (3)
N6—Co1—N1—C1	85.4 (3)	N9—Co2—N12—O8	−88.8 (3)
N2—Co1—N1—C1	−6.6 (3)	N7—Co2—N12—O8	91.6 (3)
N4—Co1—N1—C1	177.4 (3)	N8—Co2—N12—O8	176.7 (3)
N5—Co1—N1—C1	−94.5 (3)	N10—Co2—N12—O8	−3.3 (3)
N6—Co1—N2—C2	−64.8 (3)	C15—N1—C1—C2	171.1 (4)
N3—Co1—N2—C2	−156.4 (3)	Co1—N1—C1—C2	−14.2 (5)
N1—Co1—N2—C2	25.8 (3)	C3—N2—C2—C1	−177.6 (4)
N5—Co1—N2—C2	115.8 (3)	Co1—N2—C2—C1	−39.4 (4)
N6—Co1—N2—C3	71.1 (3)	N1—C1—C2—N2	34.7 (5)
N3—Co1—N2—C3	−20.5 (3)	C2—N2—C3—C5	63.9 (5)
N1—Co1—N2—C3	161.6 (3)	Co1—N2—C3—C5	−69.1 (4)
N5—Co1—N2—C3	−108.3 (3)	C2—N2—C3—C4	−58.5 (5)
N6—Co1—N3—C7	−101.9 (4)	Co1—N2—C3—C4	168.4 (3)
N2—Co1—N3—C7	−9.8 (4)	C2—N2—C3—C6	−173.3 (3)
N4—Co1—N3—C7	166.2 (4)	Co1—N2—C3—C6	53.6 (4)
N5—Co1—N3—C7	78.0 (4)	N2—C3—C6—C7	−64.8 (5)
N6—Co1—N3—C9	84.9 (3)	C5—C3—C6—C7	59.5 (5)
N2—Co1—N3—C9	177.1 (3)	C4—C3—C6—C7	179.2 (4)
N4—Co1—N3—C9	−6.9 (3)	C9—N3—C7—C6	174.8 (4)
N5—Co1—N3—C9	−95.2 (3)	Co1—N3—C7—C6	2.0 (6)
N6—Co1—N4—C10	−63.3 (3)	C9—N3—C7—C8	−4.2 (6)
N3—Co1—N4—C10	27.9 (3)	Co1—N3—C7—C8	−177.0 (3)
N1—Co1—N4—C10	−154.3 (3)	C3—C6—C7—N3	38.4 (6)
N5—Co1—N4—C10	116.0 (3)	C3—C6—C7—C8	−142.6 (4)
N6—Co1—N4—C11	72.3 (3)	C7—N3—C9—C10	170.6 (4)
N3—Co1—N4—C11	163.5 (3)	Co1—N3—C9—C10	−15.6 (4)
N1—Co1—N4—C11	−18.6 (3)	C11—N4—C10—C9	179.9 (3)
N5—Co1—N4—C11	−108.4 (3)	Co1—N4—C10—C9	−41.9 (4)
N3—Co1—N5—O2	−101.9 (3)	N3—C9—C10—N4	37.1 (5)
N1—Co1—N5—O2	78.1 (3)	C10—N4—C11—C12	62.9 (4)
N2—Co1—N5—O2	−7.0 (3)	Co1—N4—C11—C12	−70.2 (4)
N4—Co1—N5—O2	173.6 (3)	C10—N4—C11—C13	−60.8 (4)
N3—Co1—N5—O1	74.7 (3)	Co1—N4—C11—C13	166.0 (3)
N1—Co1—N5—O1	−105.3 (3)	C10—N4—C11—C14	−175.7 (3)
N2—Co1—N5—O1	169.6 (3)	Co1—N4—C11—C14	51.2 (4)
N4—Co1—N5—O1	−9.8 (3)	N4—C11—C14—C15	−65.6 (5)
N3—Co1—N6—O4	−20.9 (4)	C12—C11—C14—C15	58.0 (5)
N1—Co1—N6—O4	159.1 (4)	C13—C11—C14—C15	178.2 (4)
N2—Co1—N6—O4	−115.8 (3)	C1—N1—C15—C16	−7.1 (6)
N4—Co1—N6—O4	63.6 (4)	Co1—N1—C15—C16	179.1 (3)
N3—Co1—N6—O3	158.0 (3)	C1—N1—C15—C14	172.1 (4)
N1—Co1—N6—O3	−22.0 (3)	Co1—N1—C15—C14	−1.8 (6)
N2—Co1—N6—O3	63.1 (4)	C11—C14—C15—N1	42.1 (6)
N4—Co1—N6—O3	−117.5 (3)	C11—C14—C15—C16	−138.7 (4)
N11—Co2—N7—C31	98.5 (4)	C31—N7—C17—C18	−168.8 (4)
N8—Co2—N7—C31	−170.1 (4)	Co2—N7—C17—C18	15.4 (5)
N10—Co2—N7—C31	6.7 (4)	C19—N8—C18—C17	178.1 (4)
N12—Co2—N7—C31	−81.1 (4)	Co2—N8—C18—C17	38.7 (4)
N11—Co2—N7—C17	−86.2 (3)	N7—C17—C18—N8	−34.8 (5)
N8—Co2—N7—C17	5.2 (3)	C18—N8—C19—C22	172.0 (4)
N10—Co2—N7—C17	−178.0 (3)	Co2—N8—C19—C22	−53.2 (4)
N12—Co2—N7—C17	94.2 (3)	C18—N8—C19—C21	−66.0 (5)
N9—Co2—N8—C18	158.8 (3)	Co2—N8—C19—C21	68.8 (5)
N11—Co2—N8—C18	66.6 (3)	C18—N8—C19—C20	54.6 (5)
N7—Co2—N8—C18	−25.1 (3)	Co2—N8—C19—C20	−170.6 (3)
N12—Co2—N8—C18	−112.1 (3)	N8—C19—C22—C23	61.8 (5)
N9—Co2—N8—C19	22.1 (4)	C21—C19—C22—C23	−61.0 (5)
N11—Co2—N8—C19	−70.1 (4)	C20—C19—C22—C23	179.7 (4)
N7—Co2—N8—C19	−161.8 (4)	C25—N9—C23—C22	−173.8 (4)
N12—Co2—N8—C19	111.2 (4)	Co2—N9—C23—C22	−1.5 (7)
N11—Co2—N9—C23	99.1 (4)	C25—N9—C23—C24	2.1 (6)
N8—Co2—N9—C23	7.4 (4)	Co2—N9—C23—C24	174.4 (3)
N10—Co2—N9—C23	−169.3 (4)	C19—C22—C23—N9	−35.8 (6)
N12—Co2—N9—C23	−81.3 (4)	C19—C22—C23—C24	148.0 (4)
N11—Co2—N9—C25	−88.1 (3)	C23—N9—C25—C26	−167.9 (4)
N8—Co2—N9—C25	−179.8 (3)	Co2—N9—C25—C26	18.6 (5)
N10—Co2—N9—C25	3.4 (3)	C27—N10—C26—C25	179.4 (4)
N12—Co2—N9—C25	91.5 (3)	Co2—N10—C26—C25	40.4 (4)
N9—Co2—N10—C26	−25.1 (3)	N9—C25—C26—N10	−38.2 (5)
N11—Co2—N10—C26	66.9 (3)	C26—N10—C27—C30	172.4 (4)
N7—Co2—N10—C26	158.8 (3)	Co2—N10—C27—C30	−53.0 (4)
N12—Co2—N10—C26	−114.5 (3)	C26—N10—C27—C28	−65.7 (5)
N9—Co2—N10—C27	−162.4 (3)	Co2—N10—C27—C28	68.9 (5)
N11—Co2—N10—C27	−70.5 (3)	C26—N10—C27—C29	54.4 (5)
N7—Co2—N10—C27	21.5 (3)	Co2—N10—C27—C29	−171.0 (3)
N12—Co2—N10—C27	108.2 (3)	N10—C27—C30—C31	62.2 (5)
N9—Co2—N11—O5	−156.3 (3)	C28—C27—C30—C31	−61.7 (5)
N7—Co2—N11—O5	23.3 (3)	C29—C27—C30—C31	−179.5 (4)
N8—Co2—N11—O5	−61.7 (3)	C17—N7—C31—C32	2.9 (7)
N10—Co2—N11—O5	118.2 (3)	Co2—N7—C31—C32	178.0 (3)
N9—Co2—N11—O6	24.7 (4)	C17—N7—C31—C30	−175.4 (4)
N7—Co2—N11—O6	−155.7 (3)	Co2—N7—C31—C30	−0.3 (7)
N8—Co2—N11—O6	119.3 (3)	C27—C30—C31—N7	−36.5 (7)
N10—Co2—N11—O6	−60.8 (3)	C27—C30—C31—C32	145.1 (4)

### Hydrogen-bond geometry (Å, °)

**Table:**

*D*—H···*A*	*D*—H	H···*A*	*D*···*A*	*D*—H···*A*
N2—H2···O2	0.88	2.02	2.718 (5)	135
N4—H4···O1	0.88	2.02	2.718 (5)	135
N8—H8···O7	0.88	2.04	2.744 (5)	136
N10—H10···O8	0.88	2.03	2.732 (5)	136
O1W—H1W···O9	0.85	2.24	2.907 (7)	135
O1W—H2W···O10^i^	0.85	2.34	2.988 (7)	134
C1—H1A···O6	0.99	2.46	3.428 (6)	166
C6—H6B···O13^ii^	0.99	2.45	3.426 (6)	169
C8—H8A···O2^i^	0.98	2.53	3.352 (6)	141
C8—H8C···O7^iii^	0.98	2.58	3.549 (6)	169
C9—H9A···O1^i^	0.99	2.53	3.441 (6)	153
C10—H10A···O1W^i^	0.99	2.59	3.115 (8)	114
C10—H10A···O10^i^	0.99	2.59	3.256 (6)	125
C16—H16A···O3^iv^	0.98	2.46	3.384 (6)	157
C16—H16B···O6	0.98	2.53	3.329 (6)	138
C17—H17B···O7^i^	0.99	2.59	3.426 (6)	142
C18—H18B···O10^v^	0.99	2.53	3.230 (6)	128
C20—H20A···O12^vi^	0.98	2.57	3.424 (7)	145
C21—H21B···O12^vii^	0.98	2.57	3.535 (6)	167
C24—H24A···O6^iv^	0.98	2.53	3.401 (6)	148
C25—H25B···O3^iv^	0.99	2.47	3.421 (6)	161
C26—H26B···O15	0.99	2.37	3.262 (6)	149
C28—H28B···O14^i^	0.98	2.56	3.264 (6)	129
C32—H32B···O14^viii^	0.98	2.45	3.384 (6)	159

Symmetry codes: (i) *x*, *y*−1, *z*; (ii) −*x*+1, *y*−3/2, −*z*+1; (iii) *x*+1, *y*−1, *z*; (iv) *x*, *y*+1, *z*; (v) *x*−1, *y*−1, *z*; (vi) *x*−1, *y*, *z*; (vii) −*x*+1, *y*−1/2, −*z*; (viii) −*x*, *y*−1/2, −*z*+1.
